# Ultra-broadband axicon transducer for optoacoustic endoscopy

**DOI:** 10.1038/s41598-021-81117-7

**Published:** 2021-01-18

**Authors:** Zakiullah Ali, Christian Zakian, Vasilis Ntziachristos

**Affiliations:** 1 Chair of Biological Imaging, Central Institute for Translational Cancer Research (TranslaTUM), Technical University of Munich, Munich, Germany; 2 Institute of Biological and Medical Imaging, Helmholtz Zentrum München, Neuherberg, Germany

**Keywords:** Biomedical engineering, Imaging and sensing

## Abstract

Image performance in optoacoustic endoscopy depends markedly on the design of the transducer employed. Ideally, high-resolution performance is required over an expanded depth of focus. Current optoacoustic focused transducers achieve lateral resolutions in the range of tens of microns in the mesoscopic regime, but their depth of focus is limited to hundreds of microns by the nature of their spherical geometry. We designed an ultra-broadband axicon detector with a 2 mm central aperture and investigated whether the imaging characteristics exceeded those of a spherical detector of similar size. We show a previously undocumented ability to achieve a broadband elongated pencil-beam optoacoustic sensitivity with an axicon detection geometry, providing approximately 40 μm-lateral resolution maintained over a depth of focus of 950 μm—3.8 times that of the reference spherical detector. This performance could potentially lead to optoacoustic endoscopes that can visualize optical absorption deeper and with higher resolution than any other optical endoscope today.

## Introduction

Optoacoustic mesoscopy operating at ultra-wideband (UWB) ultrasound frequencies offers unique imaging characteristics by detecting optical absorption through several millimeters of depth with resolutions in the range of tens of micrometers^1,2^. This performance has been implemented as raster scanning optoacoustic mesoscopy (RSOM) to yield detailed visualization of different skin features, including dermal vasculature^3,4^ or melanoma related angiogenesis^5^, and the label-free quantification of inflammation associated with psoriasis, eczema or vasculitis^2^. RSOM utilizes tomographic principles by employing detectors with large acceptance angles and combining signals acquired over different projections by mathematical inversion to produce highly detailed images of tissue.

Mesoscopic performance could also be useful in gastrointestinal (GI) endoscopic applications to yield similar sub-surface visualization of optical contrast associated with vascularization and angiogenesis, possibly leading to better staging of cancer. For example, the average healthy esophagus wall is 5.26 mm thick for males and 4.34 mm thick for females^6^ and is highly vascularized, with vessels within the mucosa ranging from tens to several hundreds of micrometers in size^7^. This variation in vascular size and depth yields an optoacoustic frequency content that is inherently broadband, requiring ultrasound detectors with central frequencies up to 50 MHz and bandwidths ranging from a few to 100 MHz to provide reasonable resolution of esophageal layers^8^. Therefore, similarly to RSOM, broadband detectors are required for detailed optoacoustic imaging of the gastrointestinal wall. However, optoacoustic endoscopy has two major differences compared to RSOM. First, imaging hollow organs typically necessitates designs that rotate instead of raster scan the detector, possibly requiring transducer designs with different specifications compared to those employed in RSOM. Second, in contrast to using tomographic imaging, which can synthetically reach high imaging performance, rotational endoscopy does not collect highly overlapping projections due to the geometry of the outward looking detector. Therefore, image performance largely depends on the specifications of the detector used. Moreover, endoscopes are further limited by the requirement of miniaturized form factors to achieve sizes appropriate for insertion through cavities and hollow structures.

Over the last two decades, a number of optoacoustic endoscope design approaches have been proposed for gastrointestinal applications. These aimed to either miniaturize the probes to fit the working channel of existing white light endoscopes (WLE) or to operate as stand-alone tethered encapsulated probes. A 4.2 mm diameter probe was proposed, comprising an acoustic 45-degree reflector in front of a 43 MHz unfocused transducer with a 2 mm central aperture^9^. This approach was further enhanced by introducing a spherically focused transducer into a miniaturized 2.5 mm-diameter probe to improve lateral resolution^10^. In contrast, He et al. proposed the use of 15, 20 and 50 MHz unfocused side-looking transducers that fit into a 3.6 mm diameter probe to image the mucosal wall directly, without the need for reflectors^8,11^, similar to optoacoustic probes used for intra-vascular applications^12–15^. Optical ultrasound detectors operating at 20 MHz have also been employed for endoscopic applications; however, only for forward looking configurations^16^. Recently, our group implemented a 20 mm diameter stand-alone capsule optoacoustic endoscope, which featured a 30 MHz side-looking focused transducer with an opening for central illumination^17^. This endoscope could be oriented directly toward the organ wall to avoid indirect signal detection through reflective elements and reduce the propagation distance to improve acoustic sensitivity. A 7 mm diameter capsule-shaped probe with a 50 MHz-focused detector was applied to characterize intestinal strictures caused by Crohn’s disease in New Zealand white rabbits^18^, but the sensitivity of the sensor was limited, hence 30 measurements had to be averaged to overcome the noise.

The abovementioned developments in optoacoustic endoscopes have been largely centered around the bandwidths and frequencies of focused spherical transducers. Fine-tuning these characteristics has enabled lateral resolutions in the range of tens of microns in the mesoscopic regime. However, the depth of focus of these transducers is inherently limited to hundreds of microns by their spherical geometry. Resolving depth information with spherically focused transducers therefore requires computationally intensive reconstruction algorithms, such as back projection^19^ and model-based^20^ methods, which limits the ability to achieve high quality real-time volumetric imaging.

In contrast, axicon transducers comprise conical lenses, which focus ultrasound waves along the axial direction. This configuration yields a pencil beam sensitivity profile with high lateral resolution over an extended depth of focus. We therefore hypothesized that the elongated sensitivity profiles of axicon detectors could enable reconstruction-free image processing over greater depths than spherical detectors for optoacoustic applications. Although elongated sensitivity profiles have been investigated for optical^21^ and ultrasound imaging^22,23^, only one report on single-element transducers for optoacoustics was explored in 2009^24^, which employed lower central frequencies and bandwidths than those desirable for optoacoustic mesosocopy.

To achieve high lateral resolutions over a large depth of focus, we produced an ultra-broadband axicon optoacoustic detector. By scanning the transducer around a point source in 3D space, we are able to measure the total impulse response and provide spatial impulse sensitivity maps. We compared the performance of single element transducers with axicon and spherical geometries, both of which had central apertures required for applications with central optoacoustic illumination. Furthermore, the attributes of these detectors were measured against the focused transducer commonly used in RSOM^2^. We demonstrate for the first time a broadband pencil-beam optoacoustic sensitivity detector field, which could be used for optoacoustic mesoscopic and endoscopic applications with greater depth of focus.

## Methods

**Figure 1:**
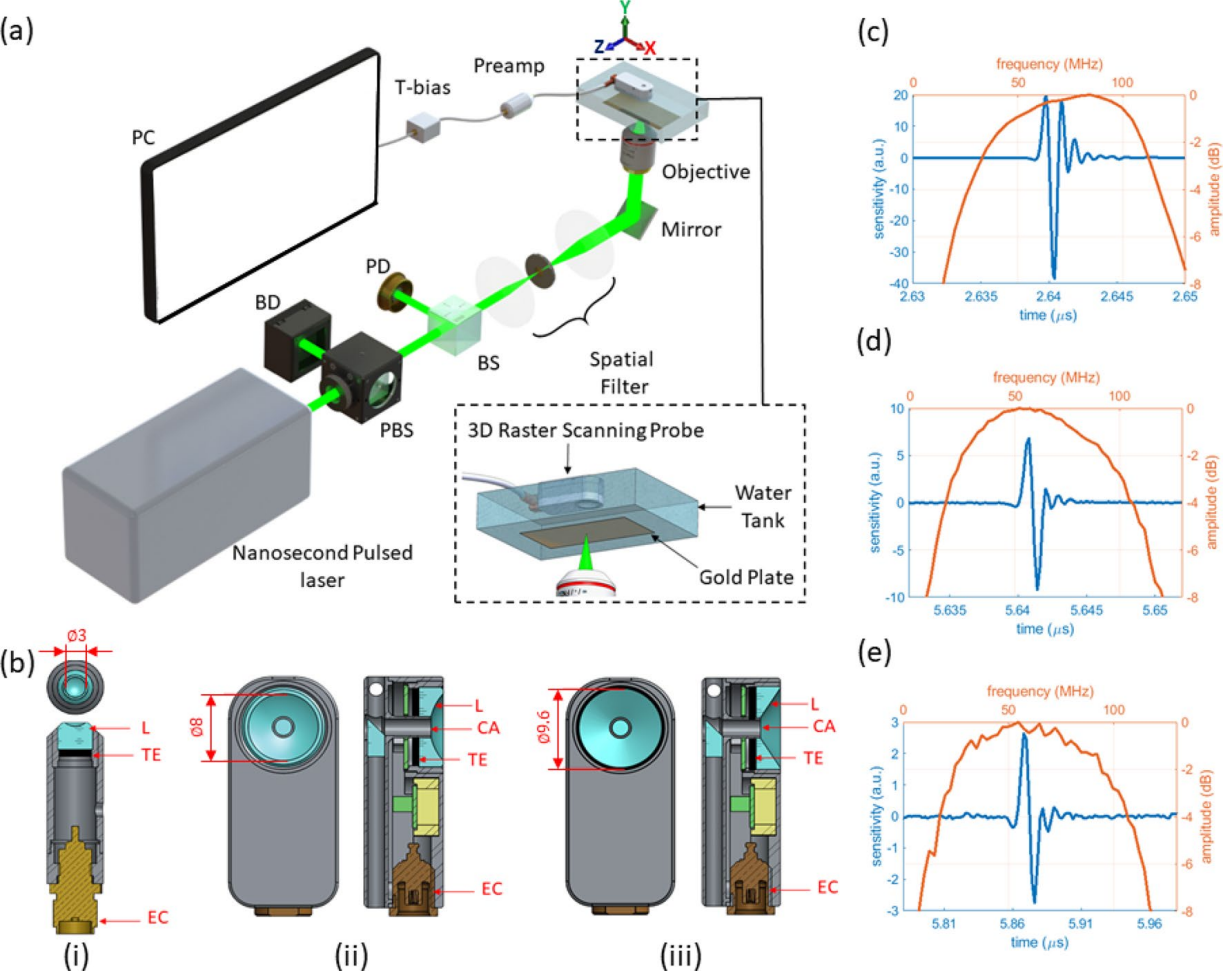
(**a**) Ultra-broadband single element ultrasound transducer characterization system used to measure the total impulse response. PBS: polarizing beam splitter, BS: beam splitter, BD: beam dump, PD: photodiode. (**b**) Lithium niobate (LiNbO_3_) optoacoustic transducer cross-section and en-face views. (i) HFM23 spherical transducer (no hole). (ii) HFM36 spherical transducer with central aperture. (iii) HFM37 axicon through with central aperture. TE: Transducer element, EC: Electrical Connector, L: Acoustic Lens, CA: Central Aperture Window. (**c**–**e**) Optoacoustic transducer temporal and frequency responses of (**c**) HFM23, (**d**) HFM36 and (**e**) HFM37 sensors. Transducer drawings in (**b**) adapted from CAD models courtesy of Sonaxis SA.

Three broadband single element optoacoustic detectors were manufactured, including a standard RSOM transducer^2^ and two endoscopic transducers with dimensions suitable for capsule endoscopy^17^. The frequency bandwidths were designed to detect signals from a few MHz up to a 100 MHz to resolve the vascular layers in the esophagus wall with sufficient sensitivity^8^. All detectors featured a planar lithium niobate (LiNbO_3_) active element with acoustic focusing achieved by mounting a customized fused silica acoustic lens on the sensor. The first transducer featured a 3 mm acoustic lens without a central aperture previously deployed for RSOM (HFM23, Sonaxis SA, France). The second and third detectors were fabricated for side-looking endoscopic applications including a 2 mm central aperture suitable for central illumination and fitted with a spherically (HFM36, Sonaxis SA, France) and conically (HFM37, Sonaxis SA, France) focused acoustic lens, respectively. The influence of a central opening is determined by comparing the performance of the spherically focused transducers, HFM23 and HFM36, whereas the impact on the depth of focus is established by comparing the sensitivity fields of the transducers with central opening but different lens shapes, HFM36 and HFM37. Figure 1b depicts 3D schematics of the transducers developed with main elements highlighted. Figure 1b(i) illustrates a spherical transducer previously utilized for RSOM (HFM23, Sonaxis SA, France) with acoustic lens of 3 mm diameter and female SMA connector. Figure 1b(ii) and (iii) depict the side-looking transducers with the spherically (HFM36, Sonaxis SA, France) and conically (HFM37, Sonaxis SA, France) focused lens, respectively. The spherical transducer was manufactured with a diameter of 8 mm and the conical transducer had a 114.4 $^\circ$ apex angle with diameter of 9.6 mm; both with a 2 mm-central hole and MCX female electrical connectors.

An inverted microscope was designed to characterize the optoacoustic response of broadband transducers as depicted in Fig. 1a. The characterization unit consists of a 1 ns-pulse width, 532 nm light source operating at 2 kHz repetition rate (Wedge HB532, Bright Solutions, Italy). The energy per pulse was regulated by means of a polarizing beam splitter and a beam dump. A 90:10 beam splitter was employed to divert 10% of the light towards a photodetector for triggering. The remaining 90% of light was spatial filtered to enhance the beam quality and focused to a spot size of approximately 12 µm with a 4X objective lens (4X Olympus RMS4X). A manual XYZ translation stage (PT3/M, Thorlabs) is utilized to align a 100 nm gold film-plate to the focal plane of the objective and generate a flat, broadband omnidirectional optoacoustic point source. The pulse energy interrogating the gold film was approximately 50 nJ.

Each transducer was mounted to an XYZ motorized stage (MTS50-Z8 in 3-Axis XYZ Configuration, Thorlabs) to perform fine linear scanning over the optoacoustic point source with deionized water employed as acoustic coupling medium. Each detector was installed with the active element facing the acoustic source and aligned with the motorized stages. A raster step scanner, using 200 signal averages per step to reduce the influence of laser pulse-to-pulse variation and background signal, was configured to sweep along the XY and XZ planes to generate the characterization maps of the sensor. The temporal optoacoustic signals were pre-amplified externally with an 30 dB amplifier (Sonaxis SA, France) and connected to a T-bias (ZFBT-4R2GW+,Mini-Circuits) prior to data acquisition. The optoacoustic data was acquired by a ATS9373 DAQ card (AlazarTech, Canada) synchronized to the board’s external trigger port via the photodetector signal. Signal processing was controlled by LabVIEW and data analyzed in MATLAB.

## Results

The designed optoacoustic characterization system enabled the evaluation of fine spatial and temporal features unique to each of the three evaluated transducer geometries, including bandwidth, focal distance, lateral and axial resolutions, and depth of focus. Figure 1c–e shows the temporal and frequency curves obtained from each transducer. The three fabricated sensors exhibit an ultra-broad bandwidth detection performance with central frequency and − 3 dB bandwidth (BW) of 72.5 MHz (BW 83 MHz), 60.5 MHz (BW 81 MHz) and 61 MHz (BW 82 MHz) for the HFM23, HFM36 and HFM37, respectively. However, the spherical transducer with no central aperture (HFM23) displays the strongest signal amplitude among the sensors at focus, yielding 4 times larger relative acoustic sensitivity (peak-to-peak signal amplitude) than the spherical with a central opening (HFM36). This is close to the 4.06 higher sensitivity expected from the relative sensor size, propagation losses to focus and absorption of the medium at central frequency. Moreover, by comparing the effect of transducer shape in the transducers with central apertures, the spherical (HFM36) revealed a 2.85 times-stronger sensitivity at focus than the axicon detector (HFM37). The focal distances and the viewing (or divergence) angles measured were 3.05 mm (52°), 7.98 mm (51°) and 8.60 mm (50°) for the HFM23, HFM36 and HFM37 transducers, respectively.

**Figure 2:**
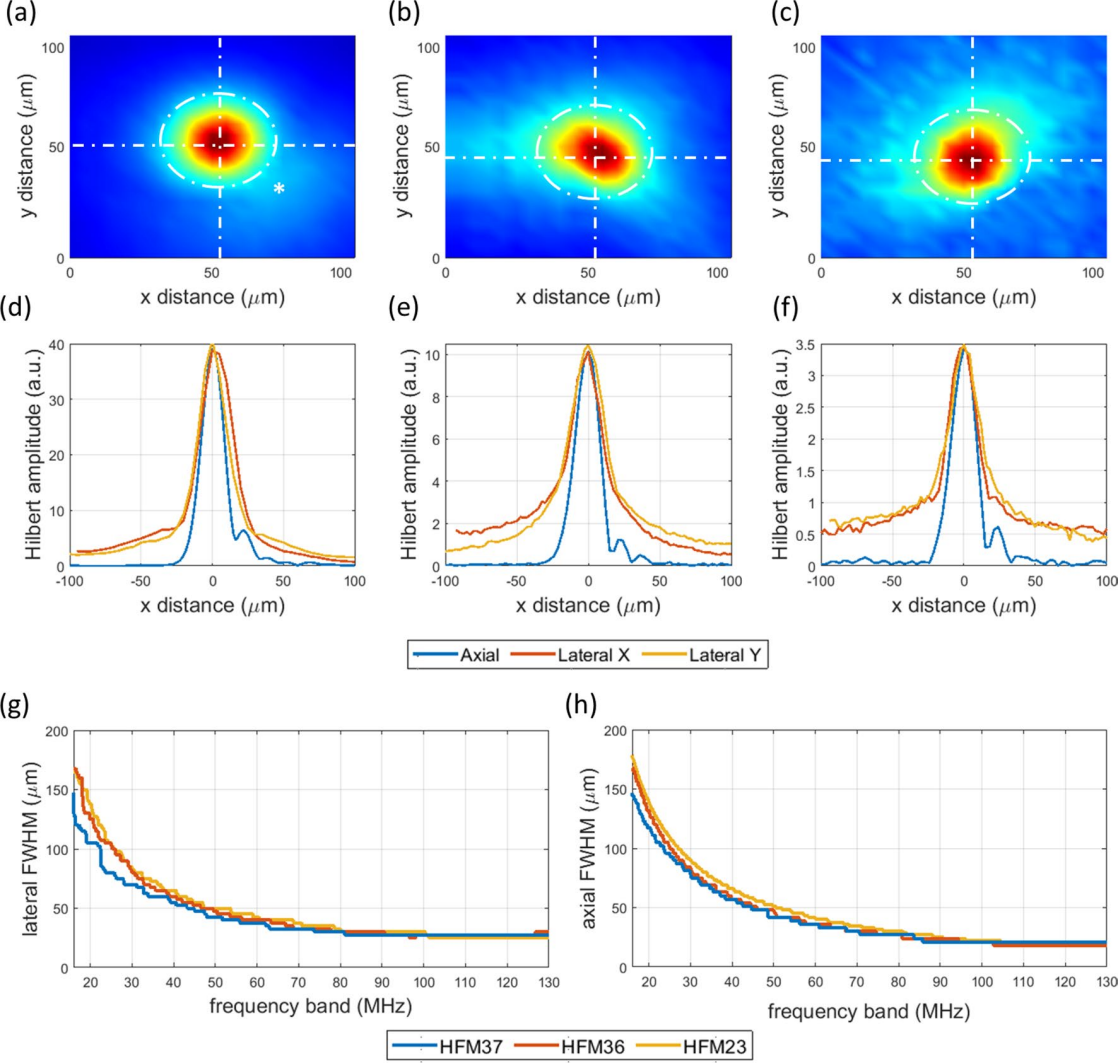
(**a**–**c**) Normalized Optoacoustic transducer sensitivity maps at XY focal planes of (**a**) HFM23 spherical transducer (no hole) (**b**) HFM36 spherical transducer with aperture (**c**) HFM37 axicon transducer with central aperture. (**d**–**f**) Axial and lateral Hilbert resolutions of each transducer respectively. (**g**, **h**) Lateral and axial resolutions as a function of transducer frequency band for all three detectors respectively.

Figure 2 shows the sensitivity maps obtained at the focal plane of each transducer. The HFM23 detector shows circular sensitivity symmetry, with a weaker secondary spot overlapping the primary one (marked in Fig. 2a). Figure 2b and c illustrate the corresponding sensitivity maps for the HFM36 and HFM37 detectors, displaying slight symmetry distortions. These astigmatic aberrations could be attributed to acoustic lens deformations during manufacturing. The lateral and axial spatial resolutions obtained for each transducer are shown in Fig. 2d–f. All three detectors yield full-bandwidth resolutions of approximately 28 μm and 18 μm, for the lateral and axial, respectively. Such a similar performance is attributed to their comparable sensitivity bandwidth. By virtue of the ultra-broad band sensitivity featured in these detectors, we further explored the dependency on acoustic bandwidth on the specific lateral resolution of each detector, as shown in Fig. 2g. An exponential relationship between resolution and frequency band was found starting from 15 MHz. The resolution limit was only obtained at full bandwidth, despite frequencies above 85 MHz having a reduced contribution to lateral resolution performance for the characteristic signal sensitivities of these transducers. This was also observed for the axial resolution, as depicted in Fig. 2h.

**Figure 3:**
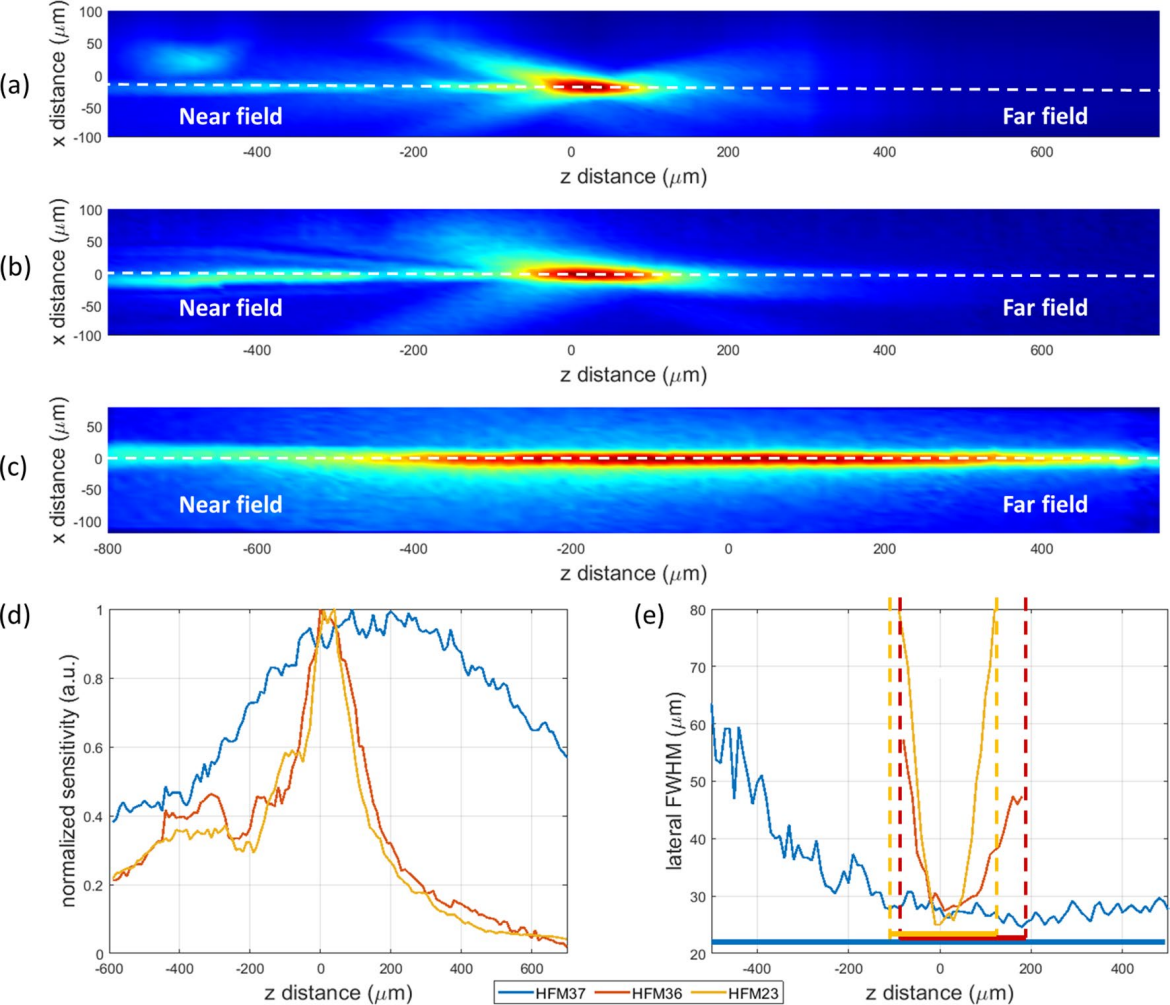
(**a**–**c**) Normalized Optoacoustic transducer sensitivity maps at XZ focal planes of (**a**) HFM23 spherical transducer (no hole) (**b**) HFM36 spherical transducer with central aperture (**c**) HFM37 axicon transducer with central aperture (**d**) Normalized depth of focus line scans of transducers depicted as dotted lines in (**a**–**c**). Note: Z = 0 corresponds to the focal point of the HFM23 and HFM36 sensors. (**e**) Lateral resolution as a function of the transducer depth of focus.

Figure 3 shows the sensitivity maps for each transducer along the (lateral) X and (axial) Z directions. In particular, the XZ sensitivity maps for the spherical detectors (HFM23 Fig. 3a, HFM36 Fig. 3b) confirm the focusing nature expected from these geometries, with low frequency lobes being observed in the near field due to the shorter propagation distance. In contrast, the XZ sensitivity map for the axicon detector (HFM37) is characterised by a thin pencil beam over an extended depth as seen in Fig. 3c. The depth of focus for all three transducers are compared in Fig. 3d, yielding 220 μm and 250 μm for the spherical cases (HFM23 and HFM36, respectively), and 1050 μm for the axicon case (HFM37)—4.2 times that of the spherical counterpart with central aperture (HFM36). As illustrated in Fig. 3e, a 40 μm lateral resolution is retained for a 950 μm depth of focus, and remains below 60 μm for a 1050 μm depth of focus. Note that the axicon detector indeed presents an extended depth compared to its spherical counterparts; however, it features lower sensitivity, in particular compared to the equivalent reference spherical detector with central aperture (Fig. 1c,e). The HMF23 measured a depth of focus approximately 30 µm smaller than the HFM36 detector, expected from the similar divergence angle measured for the spherical transducers.

## Discussion

Spherical ultrasound transducers have limited focus and lateral resolutions over extended depths. We have designed and presented here for the first time an ultra-broadband axicon ultrasound detector, which is capable of higher-resolution optoacoustic imaging than spherical detectors at depth. This axicon detector was benchmarked against a similarly sized spherical transducer of our own design with a central aperture, as well as against a smaller transducer used for optoacoustic mesoscopy that did not feature a central aperture. The improved performance of the axicon detector at depth could increase the accessible imaging depths of optoacoustic microscopes and pave the way for real time optoacoustic imaging without the need for computationally intensive image reconstruction.

Using our dedicated characterization system, we were able to demonstrate axicon ultrasound detection with broad 81 MHz-bandwidth (at a − 3 dB cut off) and extended pencil beam optoacoustic spatial sensitivity with a depth of focus of 1050 µm and a preserved 40 µm-lateral resolution over 950 µm. To our knowledge, we are the first group reporting the sensitivity performance of a high frequency broadband axicon ultrasound transducer. Several attempts to increase optoacoustic or ultrasound depth of focus at high resolution have been reported, such as incorporation of liquid lenses in optical resolution optoacoustic endoscopy^25^, multi-element electronically tunable piezoelectric sensors^26^, phase apodization techniques with concentric rings^27^, and modified back projection algorithms using synthetic aperture approaches with focused transducers^28,29^. These suffer from either restricted penetration depth, complex control algorithms, expensive fabrication procedures, large detector footprints, difficulties in miniaturize, or post processing reconstruction algorithms that increase the computational complexity limiting real-time image processing. Our results demonstrate that a broadband ultrasound detector with an axicon sensitivity field can be exploited to overcome the loss of resolution in optoacoustic imaging that is experienced at greater depths by spherical detectors.

Our sensitivity maps show that the inclusion of a central hole of up to one third of the sensor diameter in spherical detectors has a limited influence on the shape of the sensitivity map of the transducer (Fig. 3), while reducing the overall ultrasound sensitivity proportional to the area of the hole. A central aperture is advantageous for coaxial illumination, which is desirable for endoscopic applications^17^. For this reason, we designed two transducers with central apertures: an axicon transducer and a spherical transducer for comparison. In order to assess the effective change in the sensitivity field caused by the presence or absence of a hole in a transducer, we compared our designed spherical transducer (HFM36) to an established spherical detector (HFM23) with no central hole that is used for optoacoustic mesoscopy (Figs. 3a,b and 2a,b)^2^. We found that the sensitivity fields were similar for both spherical transducers. We therefore anticipate that reconstruction algorithms commonly used for spherical transducers without apertures, such as virtual point detector^28,29^ or delay multiply and sum beamforming algorithms^30,31^, could be effectively applied for either spherical transducer in this study, regardless of the central aperture. Moreover, this implies that the extended depth of focus exhibited by our axicon transducer compared to the spherical examples is independent of the axicon’s central aperture.

The high frequency broadband axicon transducer presented here could enable high-resolution optoacoustic imaging at greater depths than commonly used spherical detectors. This is particularly relevant for endoscopic applications, where tomographic reconstruction views are limited and real-time image processing is required^17^. Axicon performance is achieved solely by forming a lens in a conical rather than spherical shape in a lens-based focused transducer. Axicon spatial resolutions are achieved at full − 3 dB cut off bandwidth, and are similar to those obtained with the spherical detector. This suggests that introducing an axicon instead of spherical acoustic lens does not affect the bandwidth and resolution limit of the detector. However, a limiting factor is the trade-off between sensitivity and depth of focus. While our axicon transducer demonstrated a 4.2-fold increase in depth of focus (compared to the spherical detectors), the overall sensitivity dropped by a factor of 2.85. Additional pre-amplification could potentially compensate for some of the loss in sensitivity. Furthermore, it is possible that some sphericity was introduced into the axicon lens during the manufacturing process, as the effective theoretical 4 mm-depth of focus for our transducer design was not achieved. Future studies will focus on the integration of axicon detection into optoacoustic imaging to validate the observed performance improvements.

In conclusion, two ultra-broadband single element optoacoustic transducers have been developed and characterized. Altering the acoustic lens shape from spherical to conical allowed us to produce for the first time an 81 MHz (− 3 dB) broadband axicon detector exhibiting a pencil beam optoacoustic detection profile exceeding 1 mm depth of focus, with a retained 40 µm-lateral resolutions over a 950 µm-axial distance. Axicon profiles could potentially offer an extended penetration depth for optoacoustic microscopy and reconstruction-free approaches for optoacoustic endoscopy of the GI track and mesoscopic applications.

## Data Availability

The datasets generated during and/or analysed during the current study are available from the corresponding author on reasonable request.

## Abbreviations

UWB: Ultra-wideband
RSOM: Raster scanning optoacoustic mesoscopy
GI: Gastrointestinal
WLE: White light endoscopy
